# Study on Dry Deashing and Desulfurization of Pulverized Coal via Pulsating Circulating Airflow Technology

**DOI:** 10.3390/ma18112625

**Published:** 2025-06-04

**Authors:** Xinjian Yue, Shanshi Chen, Yongmin Zhou

**Affiliations:** College of Materials Science and Engineering, Nanjing Tech University, South Puzhu Road No. 30, Nanjing 211816, China; 202261203299@njtech.edu.cn (X.Y.); 202261203311@njtech.edu.cn (S.C.)

**Keywords:** coal separation, pulsating circulating airflow, deashing and desulfurization, pulverized coal

## Abstract

In practical coal preparation processes, influenced by mining methods and mechanization levels, the proportion of fine and even ultrafine pulverized coal continues to increase. However, due to the small particle size, significant inter-particle interactions, and the low efficiency of conventional physical separation techniques, the efficient deashing of fine coal remains a significant technical challenge. Consequently, in the face of growing demand for fine coal processing, efficient and mature dry separation technologies are still lacking. To address this issue, a pulsating circulating airflow separation device was designed and developed in this study to deash and desulfurize pulverized coal with a particle size of less than 1 mm. The effects of gas velocity and pulsating airflow frequency on the deashing performance were investigated. Using Design-Expert software (version 13), an optimized formula for deashing efficiency was established, and the optimal operating parameters were evaluated. The separation results demonstrated that under the optimal conditions of fluidization, the number N = 1.2 and pulsating airflow frequency f = 2.375 Hz, the standard deviation of ash segregation (σ_ash_) reached 25%, and the ash content in the cleaned coal was reduced from 37.28% to 22.32% in the cleaned sample. Furthermore, the sulfur content decreased significantly from 0.971% in the raw coal to 0.617% in the cleaned coal, indicating effective desulfurization. In addition, the concentrations of other harmful elements in the raw coal were also reduced to varying degrees. These findings demonstrate that the application of pulsating airflow can effectively enhance ash and sulfur removal from pulverized coal particles smaller than 1 mm. This approach offers a novel and promising method for the dry beneficiation of fine coal particles.

## 1. Introduction

Since the Industrial Revolution, coal has been a vital traditional energy source crucial to global economic development. In China, coal has long held a central position within the national energy supply system as a key energy resource and industrial fuel. In recent years, alongside the continuous strengthening of China’s economic capacity and overall national power, domestic coal consumption has surged, making China the world’s largest consumer of coal [1]. According to recent data, China’s coal production reached approximately 4.89 billion tons in 2024, marking a year-on-year increase of about 4.6%. The total annual energy consumption amounted to approximately 5.96 billion tons of standard coal equivalent, reflecting a 4.3% increase over the previous year. Coal accounted for 53.2% of the total energy consumption [2]. Simultaneously, rapid industrialization and urbanization have continued to expand domestic energy demand. As the world’s largest coal producer and consumer, coal remains the dominant energy source within China’s energy structure remains highly dependent on coal, which plays a crucial role in ensuring energy security and meeting base-load power demands. It is anticipated that coal will maintain its strategic importance for decades to come, as its role is unlikely to be entirely replaced by alternative energy sources in the foreseeable future. In addition to energy generation, recent studies have emphasized the importance of coal combustion by-products within the context of the circular economy. For instance, Ilić et al. [3] conducted a comprehensive characterization of Greek lignite bottom ash and demonstrated its potential as a raw material for construction applications due to its favorable physical and chemical properties. Such utilization pathways not only reduce the environmental impact of coal-based energy production but also add value to coal residues, offering sustainable solutions for waste management. These findings support ongoing research and industrial efforts in China to repurpose coal ash for use in cement, bricks, and road base materials, further enhancing the resource efficiency of coal utilization. Therefore, while global efforts toward renewable energy transition are accelerating, coal will continue to serve as a foundational energy source in China and many other countries for the foreseeable future.

China’s coal resources are characterized by generally poor endowment and uneven regional distribution, typified by “coal scarcity, coal abundance, low quality, and high ash and sulfur content”. In particular, reserves of high-quality coal with low ash content and high calorific value are relatively limited. In contrast, medium- to low-grade coal with high ash, sulfur, and mineral content constitutes a significant proportion of the national resource base. Currently, coal washing and processing in China still rely predominantly on wet separation technologies [4,5]. However, more than 70% of the country’s coal resources are concentrated in central and western regions—such as Shanxi and Shaanxi—with low annual precipitation, severe water scarcity, and harsh climatic conditions. In winter, average temperatures often fall below 0 °C, limiting water availability. These factors result in high costs and technical difficulties associated with wet separation processes. Moreover, coal types in these regions are primarily lignite and low-rank bituminous coal, and the ecological environment is particularly fragile [6,7]. Lignite, in particular, tends to disintegrate and form sludge upon contact with water, rendering wet separation methods highly inefficient. Therefore, there is an urgent need to develop and improve dry coal preparation technologies to enhance coal quality and utilization efficiency under challenging environmental and geological conditions [8,9].

Dry separation technology has gradually emerged as a global research focus and has attracted significant attention from various countries [10]. This technique relies on the aerodynamic effects on particles and utilizes the differences in terminal settling velocities among particles to achieve coal separation [11]. However, with the reduction in particle size and the increasing demand for high-precision separation, traditional dry separation methods have revealed certain limitations in efficiency and operational stability [12,13]. In particular, during the dry separation of fine coal particles, conventional airflow-based separation devices often suffer from intensified particle disturbances, unstable flow fields, and poorly defined separation interfaces, adversely affecting separation accuracy [14]. To address these challenges, pulsating airflow separation technology has been introduced into coal separation applications and has become the subject of active research. This approach thoroughly analyzes the enhancement mechanisms, ultimately achieving promising separation outcomes for fine coal particles [15,16]. It applies periodic external disturbances through mechanical means and generates a stable pulsating airflow with predefined frequency and amplitude. By precisely controlling pulsation parameters—such as frequency, amplitude, and waveform—the technology helps to break particle agglomerates and weaken inter-particle interactions [17,18]. This enables effective control over particle dispersion behavior and the separation interface, significantly improving the precision and efficiency of fine coal separation in airflow fields [19].

This study aims to address the ongoing challenge of efficiently separating fine coal particles (≤1 mm) by designing a pulsating circulating airflow separation system based on gas-solid flow principles under laboratory conditions. Although various dry separation methods exist, they often struggle with low efficiency or poor selectivity when applied to ultrafine or high-ash coal fractions. There remains a significant gap in achieving stable stratification and high-precision separation under unsteady airflow conditions.

In this work, A pulsating airflow separation system is designed to utilize differences in particle density and size, enabling effective stratification of pulverized coal particles and the removal of ash and sulfur by systematically adjusting airflow-related parameters such as velocity and pulsation frequency.

The findings are expected to offer novel insights and methods for de-ashing fine coal particles, providing a new approach to the efficient dry separation of small-particle-size coal. And offering a cost-effective and environmentally friendly alternative to conventional wet beneficiation methods.

## 2. Materials and Methods

### 2.1. Materials

The raw material used in this study is pulverized coal sourced from Shanxi Zhangshan Power Generation Co., Ltd, in Zhangshan, Shanxi Province, China. The power plant is located at coordinates (113.094462° E, 36.330271° N). The sample was collected between the classifier and the coal mill as returned powder.

The true density of the coal sample was determined using the alcohol pycnometer method. Approximately 20 g of dry coal powder was placed into a 50 mL pycnometer, and absolute ethanol was used as the displacement fluid to avoid absorption and ensure accurate volume measurement. The density was calculated based on the mass difference and displaced volume. The measured average true density was 1.807 g/cm^3^.

The loose bulk density was measured by gently filling a 1000 mL graduated cylinder with the coal powder without tapping or compaction. The resulting average bulk density was 0.908 g/cm^3^.

**Figure 1:**
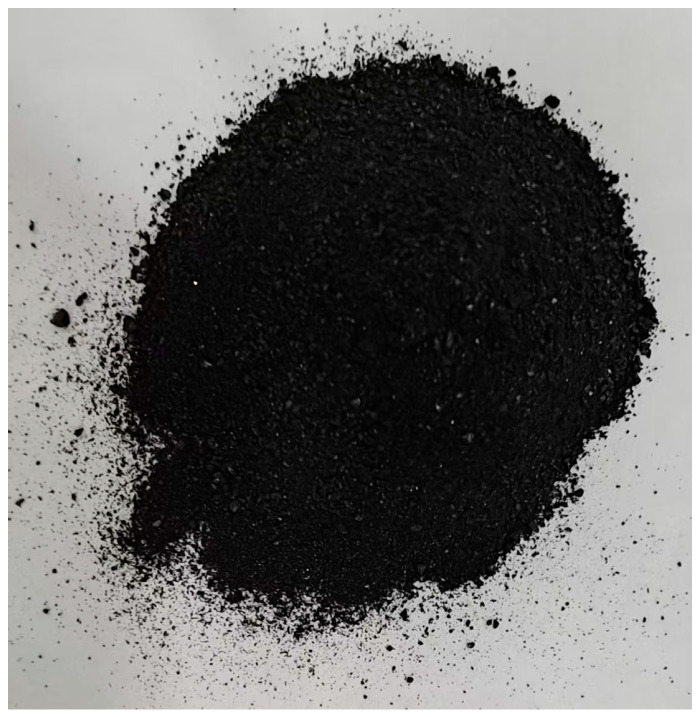
Raw material image of the pulverized coal sample.

**Figure 2:**
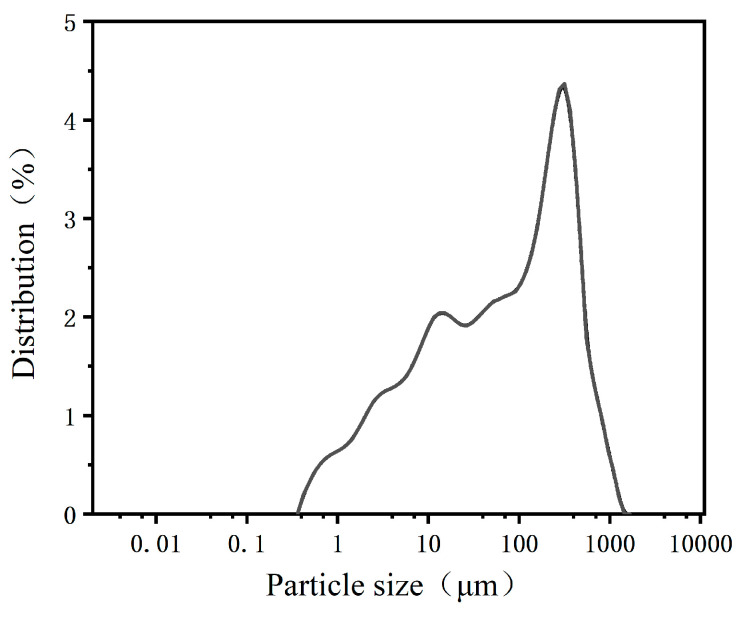
Particle size distribution of the coal sample.

Figure 1 presents an image of the raw coal sample, while Figure 2 illustrates its particle size distribution. The dominant particle size range of the sample is 100–350 μm, with an average particle diameter of 275 μm. This represents a typical fine coal sample with a relatively broad particle size distribution, though the central peak is skewed toward the finer end of the medium size range.

The proximate analysis of the coal sample, including moisture, ash, and volatile matter, was carried out following the Chinese national standard GB/T 212-2008 [20]. The true density was measured using the alcohol pycnometer method as described in Section 2.2. The detailed results are summarized in Table 1. The sample’s ash content is 37.28%, indicating a relatively high level of mineral matter, classifying it as medium—to high-ash coal. In contrast, the moisture content is relatively low, which is beneficial for maintaining good particle dispersion during the separation process.

**Figure 3:**
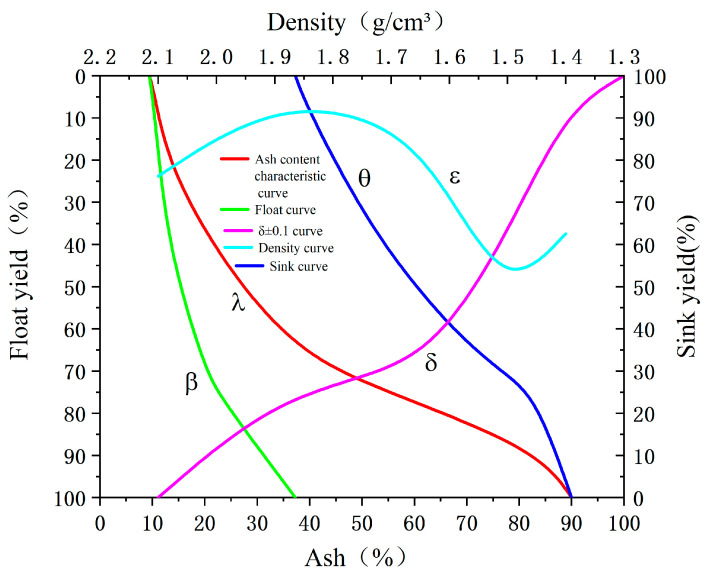
Washability curves of the pulverized coal sample.

The comprehensive results of the coal sample’s float-sink test are presented in Table 2. The test was conducted according to the Chinese national standard GB/T 478-2008 [21] (Determination of the density distribution of coal—Float and sink testing for pulverized coal). As shown in the table, approximately 60% of the sample has a density of less than 1.6 g/cm^3^, while 23.82% consists of gangue particles with densities greater than 2.0 g/cm^3^. The washability curve of the pulverized coal, shown in Figure 3, was constructed based on the float-sink test results presented in Table 2. The plotting method follows the guidelines specified in GB/T 478-2008. Indicates that effective separation can be achieved when the theoretical cut-point density lies between 1.6 and 2.0 g/cm^3^. Furthermore, the ash content varies significantly across different density fractions, suggesting that, in theory, both coal particles can be effectively separated based on density to yield fractions with different ash contents.

### 2.2. Apparatus

**Figure 4:**
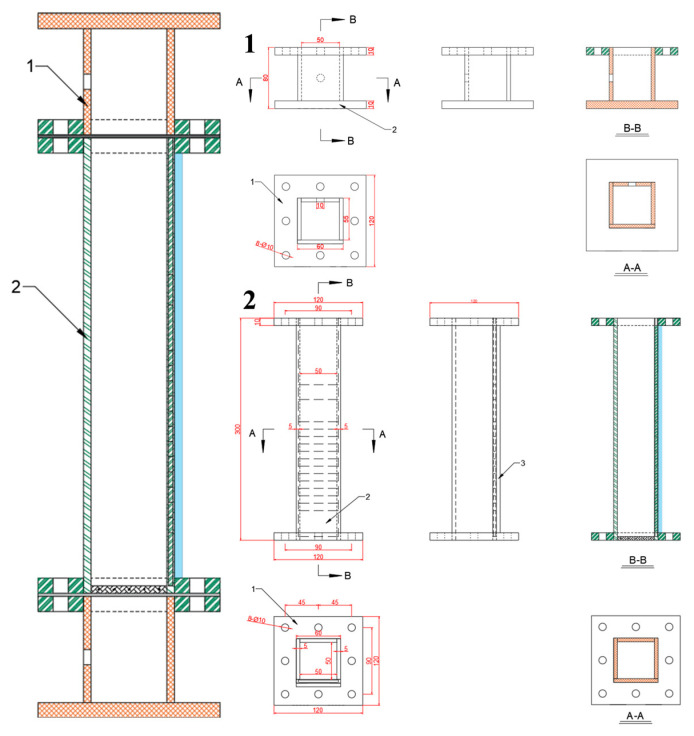
Dimensional diagram of the separation system components. In the diagram, different letters represent sectional views of the device from different directions, while the numbers indicate the detailed drawings of various parts of the device. The colored lines represent the cut sections in the sectional views of the device and are used purely for visual appeal.

**Figure 5:**
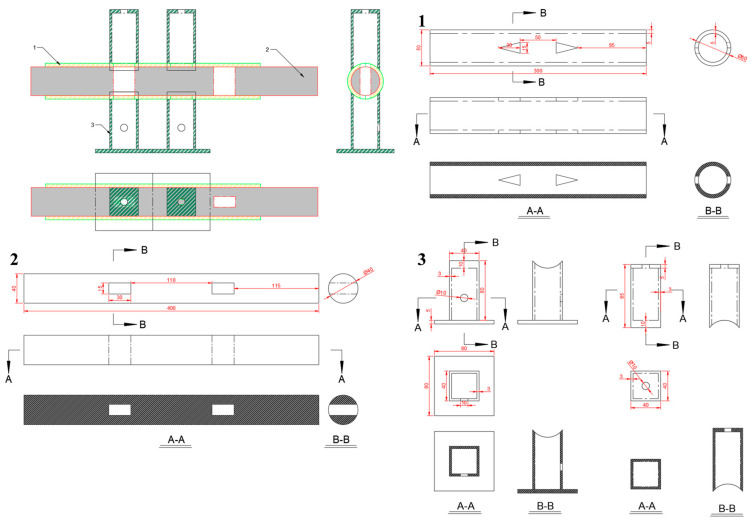
Dimensional diagram of the gas control system components. In the diagram, different letters represent sectional views of the device from different directions, while the numbers indicate the detailed drawings of various parts of the device. The colored lines represent the cut sections in the sectional views of the device and are used purely for visual appeal.

The pulsating airflow separation device used in this study was independently designed and developed. It consists of two main components: the separation system, whose dimensional schematic is shown in Figure 4, and the gas control system, illustrated in Figure 5. After the design dimensions of each component were finalized, parts were procured and assembled using a specialized acrylic adhesive to ensure an airtight structure. Since the system relies on airflow for particle separation, achieving a fully sealed assembly was essential.

A blower was used to provide a continuous gas supply, with flow regulation achieved through connected valves. A thermal gas mass flowmeter was employed to monitor the airflow rate, with a measurement range of 0.3–30 L/min. According to the manufacturer’s specifications, the flowmeter has an accuracy of approximately ±1% of full scale (F.S.).

**Figure 6:**
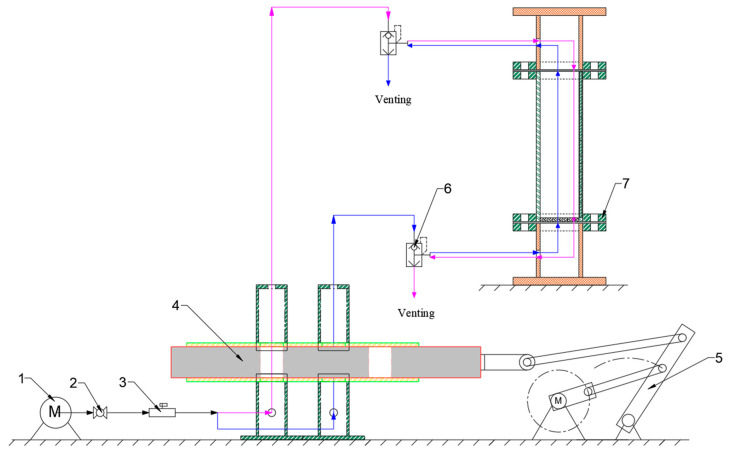
Schematic diagram of the pulsating circulation separation device: 1—blower, 2—valve, 3—flowmeter, 4—gas control system, 5—motor, 6—fast exhaust valve, 7—separator system. In the diagram, arrows and lines in different colors represent the different flow directions of gases within the device. The airflow in the gas control system is divided into two parts, each directed differently for the purpose of separation.

A schematic diagram of the entire experimental setup is shown in Figure 6. The blower continuously delivers gas, with flow rate regulated via a control valve. As the gas passes through a flowmeter, real-time flow data is acquired. The gas stream is divided into two pipelines connected to the gas circulation system. At this stage, a motor-driven reciprocating cylindrical component, controlled by remote sensing, rapidly oscillates within the device, causing continuous variation in the aperture size through which the gas passes. The motor operated at five discrete speed settings, with the corresponding measured rotational speeds being 76.5, 92.0, 113.7, 142.5, and 169.0 rpm, respectively.

When the internal cylinder moves leftward, the right-side aperture gradually opens while the left-side aperture remains closed. Gas in the right pipeline flows through this opening, passes through a fast exhaust valve, and enters the bottom air chamber of the separation unit. The gas then moves upward through the particle bed, achieving particle classification, and exits through the top air chamber via the fast exhaust valve connected to the left pipeline. Once the cylinder reaches the leftmost position, the overlapping aperture on the right side reaches its maximum. At this point, the cylinder reverses direction and moves rightward, progressively reducing the right-side aperture until completely closed.

As the cylinder continues rightward, the left-side aperture begins to open while the right-side remains sealed. Gas from the left pipeline flows through a fast exhaust valve into the top air chamber of the separator, and moves downward through the particle bed, enabling classification. The gas then passes through the bottom air chamber and exits via the fast exhaust valve on the right pipeline. When the cylinder reaches the rightmost end, the overlapping aperture on the left side reaches its maximum. The cylinder then reverses again, and the left aperture begins to close. Once fully sealed, the right-side aperture begins to reopen.

This constitutes a complete oscillation cycle, during which the pulverized coal particles are subjected to alternating directional airflow. By adjusting the motor speed and the valve opening, the flow rate, frequency, and waveform of the gas introduced into the device can be precisely controlled.

### 2.3. Methods

**Figure 7:**
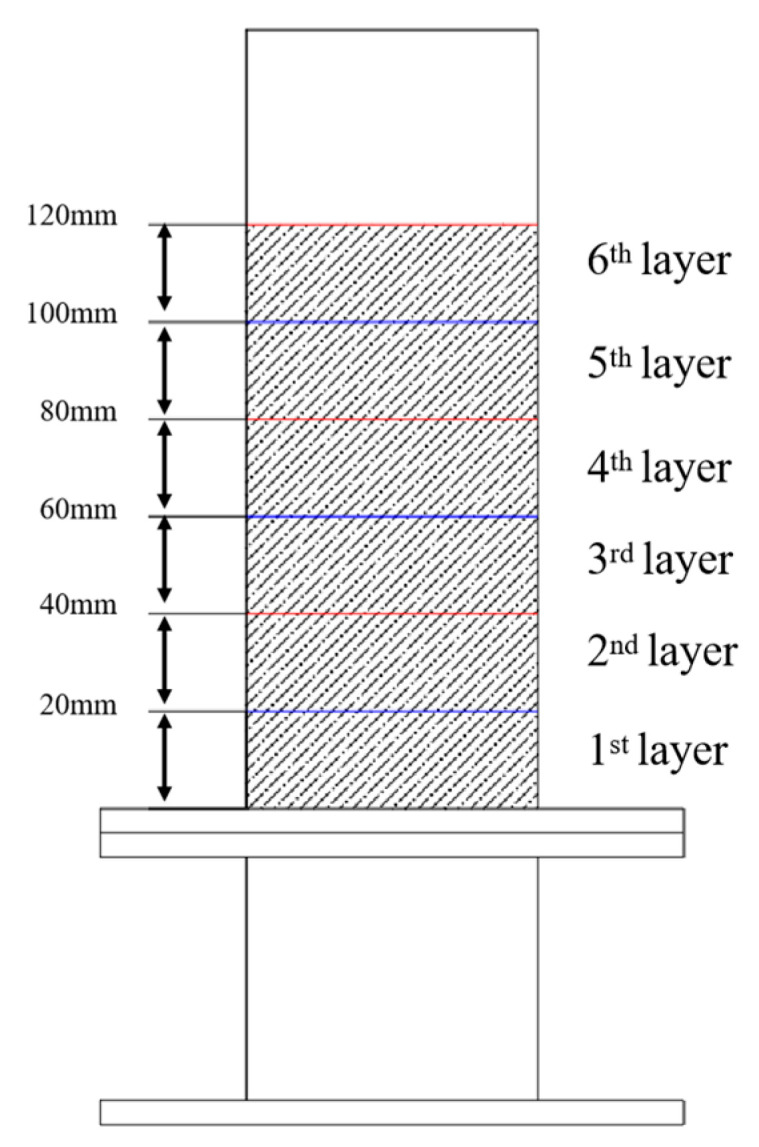
Schematic diagram of stratified sampling.

Coal particles of different densities exhibit varying ash contents, with ash content being a key indicator of coal quality and utilization value. In the separation experiments, identical pulverized coal samples were subjected to classification under different process parameters. To better evaluate the separation performance, the coal particles within the device were stratified along the *Z*-axis from bottom to top into six layers, each with a height of 20 mm. A schematic of the stratification is shown in Figure 7.

Under ambient room temperature conditions, pulsating airflow was applied for 5 min for each test condition. After separation, the samples from each layer were thoroughly mixed and then sampled to ensure the rigor and reliability of the experiment.

After each separation test, the gas input is halted, stabilizing the particles within the device. The coal samples are collected layer by layer according to the predefined stratification. The ash content of each layer is measured individually. To evaluate the degree of ash segregation within the device, the standard deviation of ash content (σ_ash_) is introduced [22]. A higher σ_ash_ indicates greater heterogeneity in the ash distribution, suggesting the presence of segregation. In this context, a larger σ_ash_ reflects a more effective density-based separation of the pulverized coal.

(1) $$\sigma_{ash}=\sqrt{\frac{1}{n-1}\sum_{i=1}^{n}{\left[A\left(i\right)-\bar{A}\right]}^{2}}$$

In the equation, *n* denotes the number of stratified sampling layers (dimensionless), *A(i)* represents the ash content of the *i*-th layer (wt.%), and *Ā* is the average ash content across all layers (wt.%).

The dimensionless fluidization number (*N*) was introduced to minimize the influence of variations in coal sample properties and particle size, enhancing the generality and comparability of the experimental results. The fluidization number is the ratio of the actual gas velocity during operation (*μ*) to the particle system’s minimum fluidization velocity (*μ_mf_*).

(2) $$N=\frac{\mu }{\mu_{mf}}$$

In the equation, *μ* represents the actual gas velocity (m/s), while *μ_mf_* denotes the minimum fluidization velocity of the pulverized coal particle system (m/s).

## 3. Results and Discussion

### 3.1. Optimization of Separation Parameters

#### 3.1.1. Effect of Gas Velocity on Separation Performance

Gas velocity is one of the most critical factors influencing the efficiency of pulverized coal de-ashing. At low gas velocities, the motion of coal particles tends to stabilize, which is unfavorable for stratification based on particle density. As a result, ash particles are difficult to separate effectively, reducing de-ashing efficiency. As the gas velocity increases, the resistance encountered by particles during separation decreases, and the centrifugal force acting on the coal particles is enhanced. This intensifies the stratification between ash and coal particles, gradually improving de-ashing efficiency. However, intense turbulence may occur when the gas velocity exceeds a certain critical threshold, resulting in particle back-mixing. This turbulence can reduce separation precision and cause previously stratified particles to remix, ultimately decreasing the overall de-ashing efficiency. Therefore, optimizing gas velocity is crucial for enhancing the efficiency of pulverized coal separation and de-ashing.

**Figure 8:**
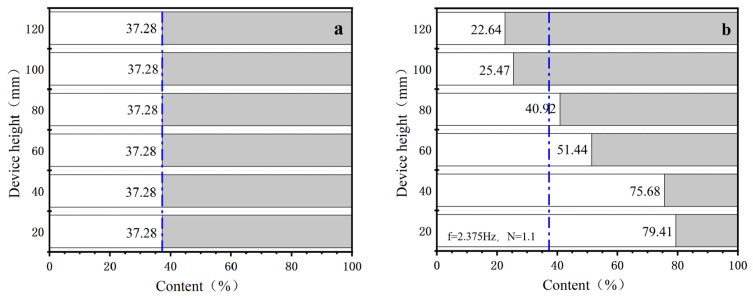

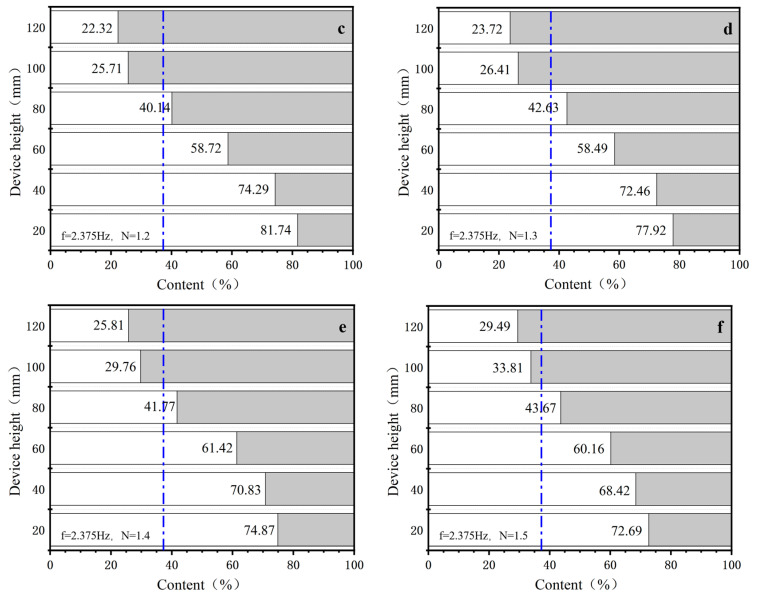
Variation in ash distribution along the height of the apparatus under different conditions: (**a**) without separation; (**b**) N = 1.1; (**c**) N = 1.2; (**d**) N = 1.3; (**e**) N = 1.4; (**f**) N = 1.5.

This study investigated the gas velocity corresponding to optimal de-ashing efficiency under a pulsating circulating airflow frequency of 2.375 Hz, using a pulverized coal fluidization number of 1.1 as the baseline. The fluidization number was gradually increased to explore its influence on de-ashing performance. In the absence of airflow intervention, coal particles of different densities are expected to be uniformly distributed within the apparatus, resulting in identical ash content at various heights, consistent with the initial ash content of the pulverized coal. Figure 8 illustrates the variation in ash content along the height of the apparatus under different gas velocity parameters. In the figure, the white portion represents the non-combustible components (i.e., ash) in the pulverized coal, the gray portion indicates the combustible fraction, and the blue double-dashed line denotes the initial ash content of the pulverized coal prior to airflow-based separation.

Experimental results from the pulsating circulating airflow separation process reveal a distinct ash content gradient within the apparatus: low-ash, low-density cleaned coal particles gradually concentrate toward the top, while high-ash, high-density gangue particles predominantly settle at the bottom. Under the operating condition of a fluidization number N = 1.2, the ash content of the coal sample at the top layer decreases to 22.32%, representing a reduction of 14.96 percentage points compared to the raw coal. Simultaneously, the ash content at the bottom increases to 81.74%, which is 44.46 percentage points higher than the raw coal’s. This indicates that the de-ashing performance is most pronounced under this condition, suggesting that N = 1.2 provides optimal separation efficiency for the pulverized coal.

As the fluidization number increases further, the de-ashing performance declines. When N = 1.5, the ash content of the cleaned coal at the top layer rises to 29.49%, only 7.79 percentage points lower than that of the raw coal, indicating the poorest de-ashing performance among the tested conditions. This suggests excessively high gas velocities disrupt particle stratification and reduce separation efficiency. In summary, as the fluidization number increases, the stratification behavior of the pulverized coal weakens, with the most favorable de-ashing performance achieved at a fluidization number of N = 1.2.

**Figure 9:**
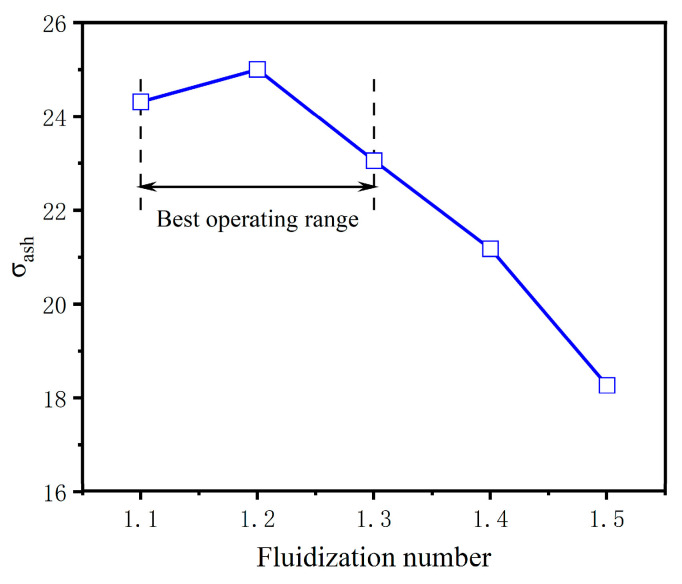
Influence of airflow velocity on σ_ash_.

Figure 9 presents the variation in the standard deviation of ash segregation (σ_ash_) as a function of gas velocity. As the velocity of the pulsating circulating airflow increases, σ_ash_ initially rises and then declines. At a fluidization number of N = 1.1, σ_ash_ is 24.31%. When N increases to 1.2, σ_ash_ reaches its maximum value of 25%, indicating the most uneven ash distribution and the most significant segregation behavior. This reflects an optimal gas velocity for enhancing ash separation, corresponding to the highest de-ashing efficiency.

With further increases in gas velocity, the separation performance begins to deteriorate, as evidenced by a progressive decrease in σ_ash_. At N = 1.5, the value of σ_ash_ drops to a minimum of 18.27%, suggesting that excessive gas velocity induces intense particle remixing within the apparatus, thereby reducing de-ashing effectiveness. Based on these findings, an optimal fluidization number range of 1.1 to 1.3 is recommended for subsequent pulsating airflow separation experiments to achieve improved separation performance.

#### 3.1.2. Effect of Circulating Airflow Frequency on Separation Performance

**Figure 10:**
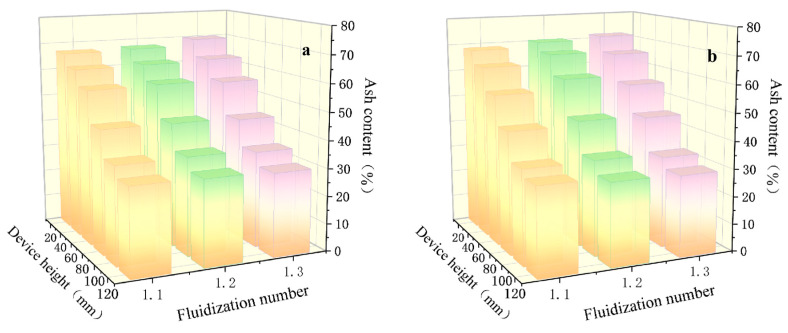

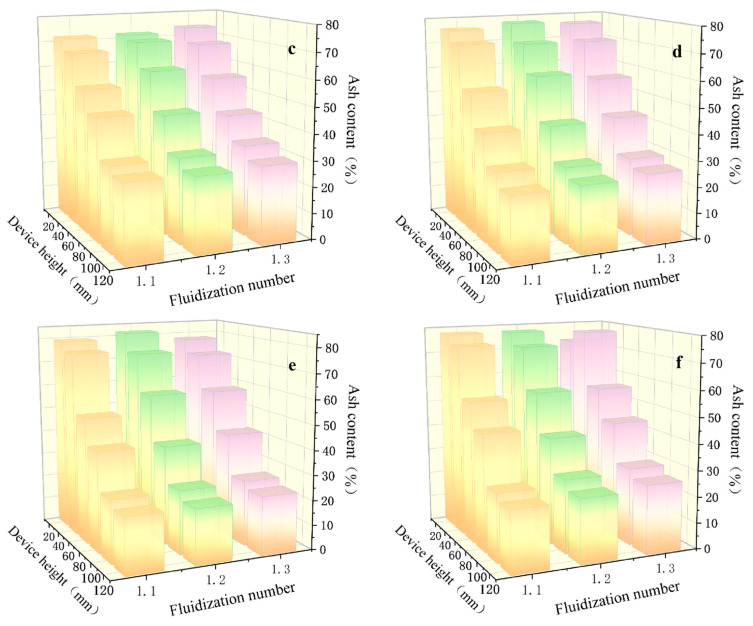
Distribution of pulverized coal ash content under different pulsating airflow frequencies: (**a**) without pulsating airflow; (**b**) f = 1.275 Hz; (**c**) f = 1.533 Hz; (**d**) f = 1.895 Hz; (**e**) f = 2.375 Hz; (**f**) f = 2.817 Hz.

Within the optimal range of fluidization numbers for de-ashing efficiency, the effect of pulsating circulating airflow frequency on the de-ashing performance of pulverized coal was investigated. The frequency of the pulsating gas flow was varied under otherwise constant conditions, with values set at 1.275 Hz, 1.533 Hz, 1.895 Hz, 2.375 Hz, and 2.817 Hz. In addition, the separation performance was compared with that of a conventional vertical continuous airflow separation device. Figure 10 illustrates the ash content distribution of pulverized coal under different frequency parameters. As shown in the figure, ash content gradually decreases with increasing height within the separation apparatus. This trend indicates that the pulverized coal undergoes stratification by density under the action of the pulsating circulating airflow, with low-density particles progressively migrating to the upper layers and high-density particles settling toward the bottom.

The trend of ash content variation exhibits noticeable differences under different gas velocities and circulating airflow conditions. Overall, higher gas velocities significantly enhance the de-ashing performance of pulverized coal under low-frequency pulsating airflow. For instance, at a pulsation frequency of 1.275 Hz and a fluidization number of N = 1.3, the ash content of the coal sample at the top of the separation column decreases to 29.54%, while the ash content at the bottom increases to 71.63%, indicating superior de-ashing performance compared to other gas velocity conditions. When the pulsation frequency is increased to 2.375 Hz, the most favorable de-ashing results are observed. Under the condition of N = 1.2, the ash content of the top-layer coal further decreases to 22.32%, while that of the bottom-layer coal rises significantly to 81.74%. Conversely, lower gas velocities demonstrate better separation efficiency at a higher pulsation frequency (f = 2.817 Hz). At N = 1.1, the ash content of the top-layer coal is reduced to 23.68%, whereas the bottom-layer ash content reaches 77.83%. These findings suggest lower gas velocities are more conducive to achieving effective particle stratification under high-frequency pulsating airflow. In summary, the synergistic interaction between appropriate gas velocity and pulsation frequency facilitates efficient density-based stratification of pulverized coal particles during separation, thereby significantly improving de-ashing efficiency.

It is worth noting that under the operating conditions of f = 2.817 Hz and N = 1.3, the ash content of the second layer of pulverized coal is significantly higher than that of the first layer after separation, indicating the occurrence of interlayer density inversion during the separation process. This abnormal distribution may be attributed to the intense changes in airflow velocity and direction inside the apparatus caused by the combined effect of high-frequency pulsation and elevated gas velocity, leading to particle remixing. As a result, some high-ash coal particles that typically settle in the lower layers become suspended in the upper-middle layers due to flow disturbances, thereby increasing the ash content of the second layer. Therefore, in the process of pulverized coal separation, it is essential to appropriately regulate the frequency of the pulsating circulating airflow to prevent remixing phenomena, thereby enhancing both de-ashing efficiency and stratification precision. Overall, the application of pulsating airflow facilitates the effective separation of low-ash and high-ash coal components, improving both the selectivity and efficiency of the separation process.

The standard deviation of ash segregation under different circulating airflow frequencies is calculated and denoted as (σ_ash_)_*p*_, representing the performance under pulsating airflow conditions. In contrast, the standard deviation of ash segregation after separation using a conventional vertical separation device under the same process conditions is denoted as (σ_ash_)_*c*_. The ratio (σ_ash_)_*p*_/(σ_ash_)_*c*_ is employed as an evaluation index to assess the enhancement in separation performance. Table 3 presents the ash segregation standard deviations under various process parameters. It can be observed that the introduction of pulsating airflow effectively improves the de-ashing efficiency of pulverized coal and optimizes the separation process. Specifically, at lower airflow frequencies, higher gas velocities lead to more pronounced improvements in separation performance. Conversely, at higher airflow frequencies, this trend reverses—lower gas velocities yield more significant improvements in the separation outcome.

**Figure 11:**
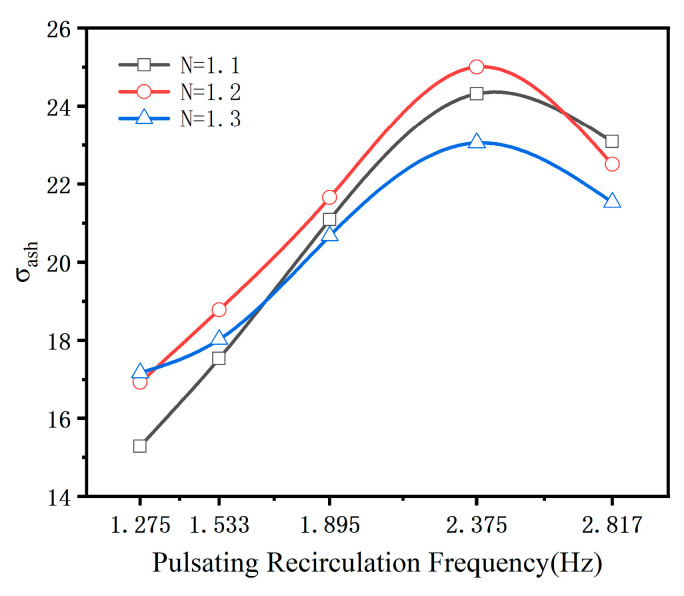
Variation trends of σ_ash_ under different process parameter conditions.

Figure 11 depicts the effect of various process parameters on the de-ashing efficiency of pulverized coal. As the pulsating circulating airflow frequency increases, the standard deviation of ash segregation (σ_ash_) under different gas velocities exhibits a general trend of increasing and decreasing. Notably, at a frequency of f = 2.375 Hz, σ_ash_ reaches its peak across all tested gas velocities, indicating the most effective separation performance. Under a fluidization number N = 1.2, the coal sample exhibits the highest de-ashing efficiency, with σ_ash_ increasing to 25%, approximately 1.682 times greater than that achieved under non-pulsating airflow conditions. When the frequency is further increased to f = 2.817 Hz, σ_ash_ decreases to 22.5%, suggesting a declining trend in de-ashing efficiency with higher pulsation frequencies. This reduction is likely due to increased particle remixing and turbulence, which hinder effective stratification.

Due to the equipment limitation that restricts the adjustment to only five discrete pulsation frequencies, the precise optimal frequency for coal de-ashing could not be definitively identified. Nevertheless, based on the current experimental results, the combination of N = 1.2 and f = 2.375 Hz is the most effective operational condition for enhancing de-ashing efficiency in the pulsating airflow separation system.

In order to verify the stability and reliability of the proposed method, triplicate experiments were performed under the optimal process parameters. As presented in Table 4, the standard deviation of ash segregation obtained from the three independent tests were 25.002%, 25.134%, and 25.252%, respectively. The standard deviation value was determined to be 0.125%. The results of the three repeated experiments under optimal conditions indicate that the proposed pulsating airflow separation system exhibits good reproducibility and operational stability.

### 3.2. Quadratic Equation of the Standard Deviation of Ash Segregation

A two-factor experimental design was conducted using Design-Expert software to evaluate the effects of fluidization number and pulsating airflow frequency on the de-ashing efficiency of pulverized coal samples. The results demonstrated that a quadratic polynomial model described the ash segregation standard deviation of coal particles smaller than 1 mm subjected to separation via pulsating circulating airflow.

The standard deviation formula of ash segregation expressed in coded factors is:

(σ_ash_) = 23.64 + 0.267 × A + 3.06 × B − 0.927 × A × B − 0.662 × A^2^ − 2.93 × B^2^

where A represents the fluidization number with a range of [−1, 1], and B denotes the pulsating circulation frequency ranging from [−1, 1].

The standard deviation formula of ash segregation expressed in terms of actual influencing factor values is:

(σ_ash_) = −133.18 + 186.138 × Q + 38.585 × E − 12.025 × Q × E − 66.196 × Q^2^ − 4.931 × E^2^

where Q represents the fluidization number, which ranges from [1.1, 1.3], and E denotes the pulsating circulation frequency, which ranges from [1.275, 2.817].

**Figure 12:**
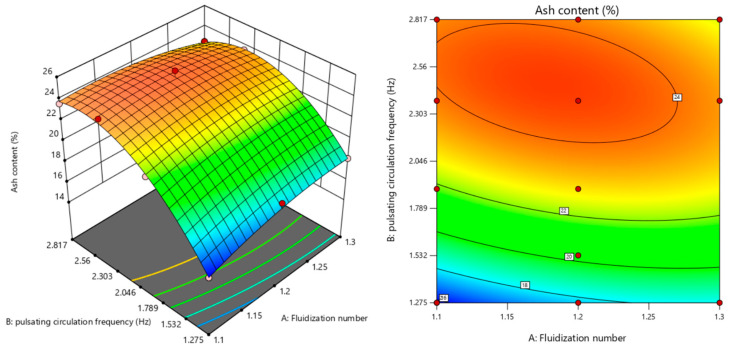
Response surface and contour plot of the fluidization number and circulation frequency interaction.

Based on the established model and calculated formulas, the optimal process parameters were determined to be a fluidization number N = 1.2 and a pulsating circulating airflow frequency f = 2.375 Hz. Under these conditions, the theoretically predicted standard deviation of ash segregation (σ_ash_) was 24.418%. In the separation process, the standard deviation under the same conditions was 25.0018%, resulting in a relative error of approximately 2.33%. This indicates the reliability of the proposed model. The interaction response surface under these conditions is shown in Figure 12.

### 3.3. Evaluation of Sulfur Removal Efficiency

**Figure 13:**
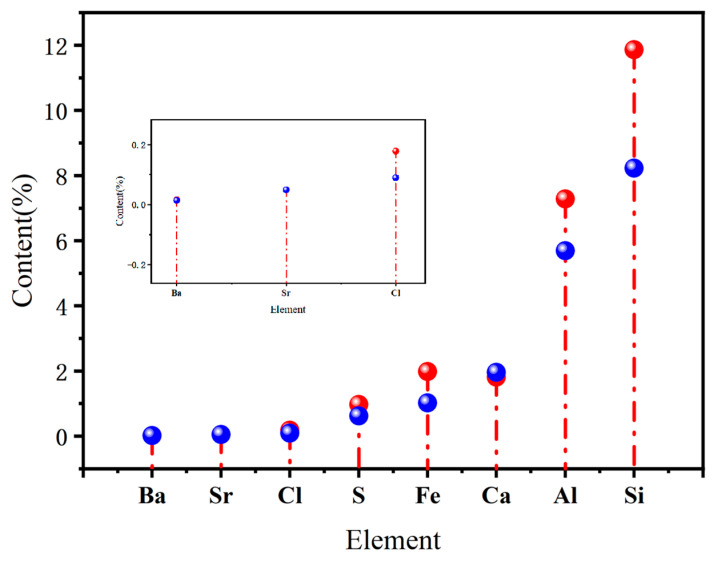
XRF analysis of raw coal and cleaned coal. The red spheres in the diagram represent the elemental composition of raw coal before separation, while the blue spheres indicate the elemental composition of clean coal after separation.

After applying the pulsating circulating airflow separation process, the experimental group exhibiting the highest de-ashing efficiency was selected for further analysis. The cleaned coal samples from the top layer after separation were sent to the key laboratory for XRF analysis. The XRF results of the raw and the cleaned coal are presented in Figure 13. The results indicate that the concentrations of elements such as Al, Si, S, and Cl were significantly reduced in the cleaned coal after separation.

The content of Al decreased from 7.28% to 5.69%, and that of Si decreased from 11.86% to 8.23%. As both Al and Si primarily exist as non-combustible minerals, their reduction contributes to lowering the proportion of inorganic impurities in pulverized coal, thereby improving its combustion efficiency. The Cl content was reduced from 0.179% to 0.09%. Although Cl is present in relatively low concentrations in coal, it can readily form gaseous hydrogen chloride (HCl) during combustion, which may lead to high-temperature chlorine-induced equipment corrosion. Therefore, reducing Cl content is important for prolonging the service life of boilers and other high-temperature facilities.

Moreover, the sulfur content decreased from 0.971% in the raw coal to 0.617% in the cleaned coal. Sulfur is one of the major hazardous elements in coal and tends to form pollutants such as SO_2_ during combustion, leading to environmental contamination and equipment corrosion. The reduction in sulfur content during the separation process helps to alleviate the burden on subsequent flue gas desulfurization systems and contributes positively to environmental protection.

In conclusion, the pulsating circulating airflow separation technology demonstrates a significant capacity for removing harmful elements from pulverized coal particles smaller than 1 mm under controlled laboratory conditions. This contributes to improving fine coal quality and providing preliminary evidence for its efficient and clean utilization.

### 3.4. Comparison of the Pulsating Airflow Separation with Other Dry Beneficiation Methods

In order to further evaluate the performance of the proposed pulsating airflow fluidized bed, our results were compared with those from other dry separation methods. For instance, Zhang et al. [23] studied a vibratory-assisted fluidized bed system for separating 6–0.5 mm coal and found that mechanical vibration combined with airflow could enhance particle stratification. However, this approach requires complex vibration modules, increased energy consumption, and presents mechanical reliability challenges in large-scale operations.

On the other hand, tribo-electrostatic separation has been widely investigated for fine coal cleaning due to its ability to distinguish particles based on surface charge differences [24]. While triboelectrostatic techniques are effective for ultra-fine particles, they are highly sensitive to ambient humidity, surface oxidation, and require strict control of material properties and environmental conditions, which limits their industrial scalability.

In contrast, the pulsating airflow generates periodic pressure waves that facilitate controlled expansion and contraction of the bed, which significantly improves the fluidization and dispersibility of fine particles. This allows even ultrafine coal powders to stratify according to density and aerodynamic behavior, while minimizing particle agglomeration and entrainment loss. Therefore, for coal particles under 1 mm, the pulsating airflow method offers a simpler, more stable, and more adaptable approach, with promising separation efficiency and scalability for dry beneficiation applications.

## 4. Conclusions

This study focuses on the de-ashing experimental investigation of pulverized coal using a specially designed pulsating circulating airflow separation device. Density classification of the coal samples was conducted through float-sink tests to determine the distribution characteristics across different density fractions, thereby providing a theoretical basis for subsequent separation experiments. The spatial distribution patterns of coal particle stratification within the separation device and the corresponding detection methods were clarified. The standard deviation of ash segregation (σ_ash_) was introduced as an evaluation metric, and the ability of pulverized coal to achieve effective density-based stratification within the separation device under different process parameter conditions was systematically investigated. The conclusions are as follows:

(1) Gas velocity and pulsating airflow frequency significantly influence the de-ashing performance of pulverized coal. After being strengthened by pulsating circulating airflow, efficient separation of coal particles smaller than 1 mm can be achieved, exhibiting a significant reduction in ash content. As the gas velocity and airflow frequency increase, the standard deviation of ash segregation (σ_ash_) shows an increasing and decreasing trend. Comprehensive analysis reveals optimal de-ashing efficiency when fluidization number N = 1.2 and the circulating airflow frequency f = 2.375 Hz is achieved. Under these conditions, a pronounced stratification is observed, with σ_ash_ reaching 25%. The ash content of the cleaned coal is reduced to 22.32%, representing a 14.96% decrease compared to the initial ash content.
(2) Based on the interaction between the fluidization number and the circulating airflow frequency, a regression model for the standard deviation of ash segregation (σ_ash_) in separating pulverized coal particles smaller than 1 mm was developed using Design-Expert experimental design software.
  (σ_ash_) = −133.18 + 186.138 × Q + 38.585 × E − 12.025 × Q × E − 66.196 × Q^2^ − 4.931 × E^2^
  The relative error between the experimentally obtained standard deviation of ash segregation under optimal process conditions and the value predicted by the model was only 2.33%, indicating that the proposed regression model is highly reliable.
(3) The pulsating circulating airflow significantly affected the removal of sulfur from pulverized coal, with the sulfur content in the cleaned coal reduced by 2.13% compared to the raw coal. Other harmful elements were also partially removed, with the contents of Si, Al, and Cl reduced by 3.63%, 1.59%, and 0.089%, respectively. These results demonstrate that the pulsating circulating airflow can effectively remove harmful elements from coal particles with a diameter of less than 1 mm.

In summary, this study systematically investigates two key process parameters—gas velocity and circulating airflow frequency—offering promising insights and practical guidance for optimizing the operation of pulsating airflow separation devices and enhancing the de-ashing efficiency of pulverized coal.

However, it is important to acknowledge the limitations of this study. The current findings are based on a laboratory-scale setup and do not yet account for factors such as energy consumption, operational costs, or long-term system stability. Moreover, no economic analysis has been conducted to assess industrial viability. Therefore, pilot-scale experiments and techno-economic analysis are needed in future work to verify the industrial feasibility and scalability of the proposed method.

## Figures and Tables

**Table 1:** Proximate Analysis of the Pulverized Coal Sample.

M_t_ (%)	A_ad_ (%)	V_daf_ (%)	FC (%)	True Density (g/cm^3^)
1.21	37.28	13.17	48.34	1.807

**Table 2:** Comprehensive Table of Floatation and Sinking Tests for Pulverized Coal Samples.

Density (g/cm^3^)	Yield (%)	Ash Content (%)	Cumulative Floats		Cumulative Sinks		Separating Density	
			Yield (%)	Ash Content (%)	Yield (%)	Ash Content (%)	Density (g/cm^3^)	Yield (%)
<1.4	8.05	10.36	8.05	10.36	100.00	37.28	1.4	37.47
1.4~1.5	29.42	13.26	37.47	12.64	91.95	39.64	1.5	51.95
1.5~1.6	22.53	25.78	60	17.57	62.53	52.05	1.6	27.74
1.6~1.8	10.42	38.17	70.42	20.62	40.00	66.85	1.7	10.42
1.8~2.0	5.76	50.85	76.18	22.91	29.58	76.95	1.9	5.76
>2.0	23.82	83.26	100	37.28	23.82	83.26	2.1	23.82
Total	100	37.28	-	-	-	-	-	-

**Table 3:** The standard deviation of ash segregation in pulverized coal under different process parameters.

	Frequency	1.275	1.533	1.895	2.375	2.817
N = 1.1	(σ_ash_)_p_	15.284	17.537	21.089	24.314	23.084
	(σ_ash_)_p_/(σ_ash_)_c_	1.028	1.18	1.419	1.636	1.553
N = 1.2	(σ_ash_)_p_	16.925	18.784	21.659	25	22.501
	(σ_ash_)_p_/(σ_ash_)_c_	1.139	1.264	1.457	1.682	1.514
N = 1.3	(σ_ash_)_p_	17.172	18.024	20.671	23.052	21.53
	(σ_ash_)_p_/(σ_ash_)_c_	1.155	1.212	1.391	1.551	1.449

**Table 4:** Results from repeated experiments conducted under optimal process conditions.

Group	Clean Coal Ash Content (%)	σ_ash_ (%)	Average (%)	SD (%)
1	22.32	25.002%	25.129	0.125
2	22.09	25.134%		
3	22.27	25.252%		

## Data Availability

The original contributions presented in the study are included in the article, further inquiries can be directed to the corresponding author.
